# Ecology and Evolutionary Biology of Hindering Phage Therapy: The Phage Tolerance vs. Phage Resistance of Bacterial Biofilms

**DOI:** 10.3390/antibiotics12020245

**Published:** 2023-01-25

**Authors:** Stephen T. Abedon

**Affiliations:** Department of Microbiology, The Ohio State University, Mansfield, OH 44906, USA; abedon.1@osu.edu

**Keywords:** bacterial self-sacrifice, bacteriophage therapy, biocontrol, biofilm matrix, phage avoidance, phage delay, phage negation

## Abstract

As with antibiotics, we can differentiate various acquired mechanisms of bacteria-mediated inhibition of the action of bacterial viruses (phages or bacteriophages) into ones of tolerance vs. resistance. These also, respectively, may be distinguished as physiological insensitivities (or protections) vs. resistance mutations, phenotypic resistance vs. genotypic resistance, temporary vs. more permanent mechanisms, and ecologically vs. also near-term evolutionarily motivated functions. These phenomena can result from multiple distinct molecular mechanisms, many of which for bacterial tolerance of phages are associated with bacterial biofilms (as is also the case for the bacterial tolerance of antibiotics). The resulting inhibitions are relevant from an applied perspective because of their potential to thwart phage-based treatments of bacterial infections, i.e., phage therapies, as well as their potential to interfere more generally with approaches to the phage-based biological control of bacterial biofilms. In other words, given the generally low toxicity of properly chosen therapeutic phages, it is a combination of phage tolerance and phage resistance, as displayed by targeted bacteria, that seems to represent the greatest impediments to phage therapy’s success. Here I explore general concepts of bacterial tolerance of vs. bacterial resistance to phages, particularly as they may be considered in association with bacterial biofilms.

## 1. Introduction

In 1941, as possible prelude to issues considered in this review, Krueger and Scribner [1] stated that “…there is no reason to assume extraordinary penetration of phage into the deeper tissues and it becomes unlikely that bacterial lysis is more than a remote possibility in anything except the very superficial areas” (p. 2163). Associated with phage-treatable infections of such body tissues can be biofilms. Biofilms are three-dimensional multi-celled structures that consist of one or more microbial species and associated extracellular polymers. The latter collectively are described as the biofilm matrix. Prominent among biofilms are those produced by bacteria, with some of these bacterial biofilms sufficiently problematic that substantial efforts are employed to prevent or remove them. Among problematic biofilms are those associated with industrial, medical, and marine biofouling [2,3], along with those contributing to persistent or chronic bacterial infections of body tissues [4,5]. These biofilms, compared to equivalent planktonic bacteria, can be somewhat impervious to small chemical-based antimicrobials. This includes being protected from eradication by the actions of both disinfectants [6,7] and antibiotics [8,9]. The result is that bacterial biofilms can cause substantial morbidity, mortality, and economic losses, but we are inadequately equipped to get rid of them. Identification of more effective alternatives to standard antimicrobial approaches to combat biofilms—including those associated with clinical bacterial infections, as is the emphasis here—therefore is desirable.

One alternative approach, which may be particularly effective against biofilms in which a single identifiable bacterial species is prominent is the use of bacterial viruses, and there is a long history of their use to treat “long-standing, chronic, or persistent bacterial infections” [10]. These viruses, commonly described as bacteriophages or phages, can naturally possess as many as four anti-biofilm properties. They are able to (1) kill bacteria; (2) lyse bacteria, including those found in biofilms [11]; (3) amplify their local activity, also in association with biofilms [12]; and, at least for some phages, (4) enzymatically degrade biofilm matrix material [13,14]. Thus, there is a potential utility of phage therapies [15,16,17] in the treatment of biofilm-associated bacterial infections [18,19].

**Figure 1:**
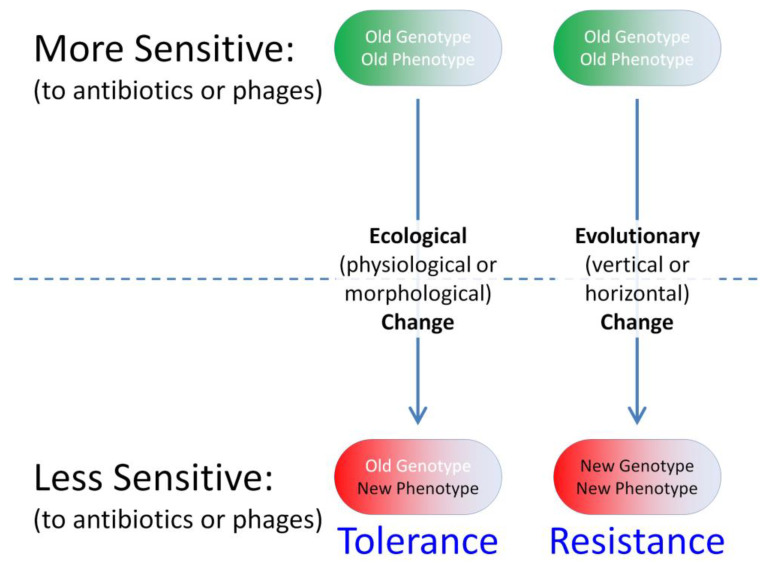
Ecological vs. evolutionary change in bacterial susceptibility to phages or antibiotics. Ecological processes involve interactions between organism phenotypes and their environments, whereas evolutionary processes involve changes in allele frequencies (organism genotypes) over time. Vertical evolutionary changes (parent to offspring) involve mutations, whereas horizontal evolutionary change (horizontal or lateral gene transfer) involves what is known as genetic migration. The latter, though not emphasized here, involves the acquisition especially of evolved genetic material from other populations, e.g., such as in the course of acquisition of plasmids by bacteria. Tolerance-type phenotypes that result from near-term evolutionary changes are also not emphasized here.

Biofilm bacteria nonetheless can oppose phage actions. This is accomplished either just phenotypically relative to planktonic bacteria, that is, without bacteria changing their genetic makeup, or instead as a consequence of genotypic changes in targeted bacterial populations, i.e., as associated with bacterial evolution (Figure 1). In the antibiotics literature, equivalent bacterial strategies are commonly dubbed as “tolerance” vs. “resistance”, respectively [4,8,20,21,22]. Tolerance of antibiotics by bacteria, in particular, represents a temporary reduction in susceptibility, one that is not observed under all circumstances and particularly not observed as measured under standard conditions in vitro. Resistance, in contrast, involves a reduced susceptibility that *is* observed during in vitro testing, i.e., in the course of minimum inhibitor concentration (MIC) determinations. 

“Tolerance” in the antibiotics literature also can be used to describe newly arising bacterial mutants that display reduced susceptibilities, except during MIC determinations. Such newly arising “tolerance”, however, is not what is being considered here; this in part is because emphasis is on non-evolutionary mechanisms of phage tolerance, and it is also because phage MICs themselves are not easily defined [23]. In any case, for reasons presumably analogous to antibiotic failures in the treatment of long-standing bacterial infections—with such infections being a typical focus of phage treatments [10,16]—phage therapies, too, are not always highly successful [24]. This suggests an importance of overcoming bacterial mechanisms of inhibition not just of antibiotic therapies but of phage therapies as well, whether that inhibition is due to a phenotypic tolerance of or instead is a consequence of an evolutionary resistance to these treatments.

An older synonym for bacterial tolerance of phages comes from Lenski [25], who postulated that “some genetically sensitive cells are physiologically insensitive” (p. 25) and which he also described as “Physiological protection”. He then went on to consider the following (p. 26; references his): “Physiological refuges may also arise as the consequence of starvation of bacteria [26], depletion of a factor in the medium required for phage adsorption [actually, there, required for DNA penetration] [27], or bacterial clumping [28]”. More recently in the phage literature, phage tolerance by bacteria has been described as a “Phenotypic resistance” [29], there emphasizing transient mechanisms of reduced bacterial adsorbability by phages such as due to reduced displays by bacteria of phage-adsorption receptors [30]. This is in contrast to simple “resistance”, though the latter could equivalently be dubbed instead as “Genetic resistance” [29,30] or a “genotypic resistance”. The latter phrasings, however, appear also to be used to describe especially antibiotic-resistance attributes that have been identified by direct analysis of DNA rather than as a synonym for simply a genetically based “resistance”. “Phenotypic resistance” alternatively can be used to describe genetically based resistance to antibiotics that is identified by phenotype rather than by sequence-based means [31,32,33]. Nevertheless, the phrase “phenotypic resistance” has been used in the phage literature at least since the early 1960s to describe various phenomena [34], including temporary (not evolutionary) mechanisms of bacterial inhibition of phage action [35]. More recently, “Phenotypic flux” also has been used to describe non-genetic physiological bases of transiently reduced bacterial sensitivities to phages [36].

Notwithstanding such precedence, here I use the term employed in the antibiotic literature of “tolerance” [37] to describe temporary mechanisms of bacterial inhibition of phages. These mechanisms as considered here are, in particular, not an immediate consequence of evolutionary change, thereby contrasting tolerance of phages also with newly acquired “resistance” to phages. The term, “tolerance”, in addition, may predate even that of “phenotypic resistance” to describe phenomena which only transiently interfere with phage antibacterial actions (Box 1). Considered in this review especially are general principles of phage tolerance and phage resistance, as these may be observed within a bacterial biofilm context. Conceptually, the phenomena described may be viewed predominantly as impacting phage display of virion production, e.g., lytic cycles, rather than affecting lysogenic cycles; however, in principle the latter can be impacted by mechanisms of phage tolerance or resistance as well.

Box 1 **Justification of use of “Phage tolerance” despite historical ambiguity in its meaning.**

**Figure 2:**
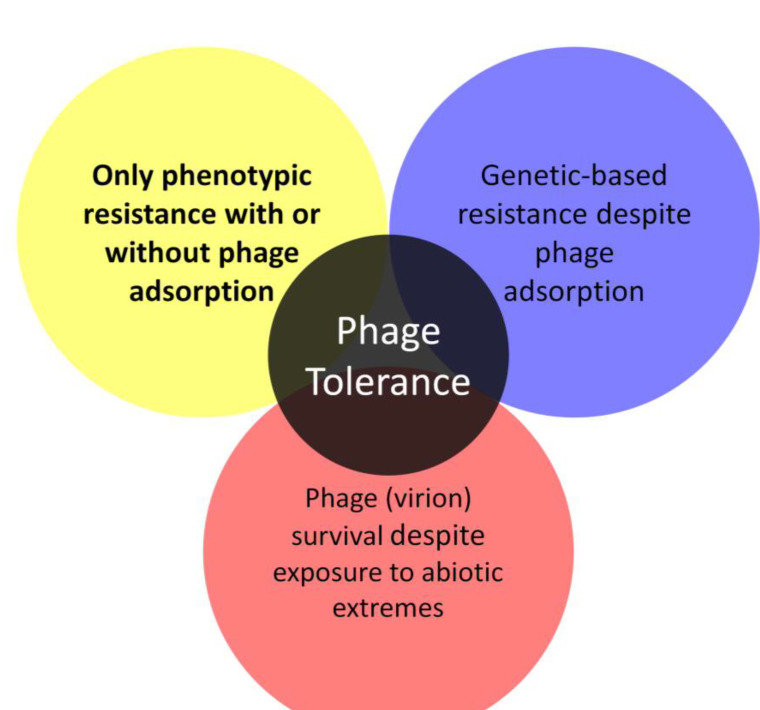
Multiple historical meanings of phage tolerance. The focus of this review is on the upper-left meaning, emphasized in bold, though with the additional meanings discussed in Box 1.

Different meanings of “Phage tolerance” have been introduced over the years (Figure 2, below). Ciofu et al. [38], in 2022, for example, used “Tolerance” to describe temporary inhibition by bacteria of phage antibacterial actions (emphases are mine here, as well as in subsequent quotations): “The reduced growth rates in the deeper biofilm layers also causes *tolerance* to lytic phages”. Henriksen et al. [39], as cited for that passage, similarly stated in 2019 (p. 9) that “…genetic mutations are important for the protection against the phage attack in our in vitro model, besides the *phage tolerance* due to microcolony formation”. Tzipilevich et al. [40], also in 2022, employed “Phage tolerance” to describe an induced mechanism of inhibition of phage action that they first observed during bacterial lawn growth, with bacterial lawn development also associated with bacterial microcolony formation [41,42,43]. See too Darch et al. [44], from 2017, who describe “increased *tolerance* to phage killing” (p. 3).

Application of the term “Tolerance” to describe inhibition of phages nevertheless is not necessarily new. Though different phrasing is possible, there are ~150 Google Scholar hits on the search, “Phage tolerance” OR “Bacteriophage tolerance”, excluding patents and citations, including 23 in 2022 (though more than one of those ~150 Google Scholar hits appears to have relatively little to do with bacteriophages). Of these, only one uses both phrases and 27 use “Bacteriophage tolerance” but not “Phage tolerance”. The earliest of these hits is that of Meanwell and Thompson [45], published in 1956. They noted (p. 292) that “…cultures with phages of slow multiplication rate should be selected as cheese starter cultures, we have found that such cultures show the highest level of *phage tolerance* before the protective action of rennet is lost”. Rennet catalyzes the coagulation or clotting of milk, thereby generating a spatially structured, semi-solid environment in which bacterial formation of biofilm-like states would presumably be encouraged. Thus, Meanwell and Thompson described a temporary inhibition of phage antibacterial activity that at least arguably is biofilm associated, though alternatively which could be a property simply of change in the physical structure of the milk rather than being due to a change in bacterial properties.

This usage by Meanwell and Thompson is followed temporally by a number of publications which do not appear to equate “Phage tolerance” with only temporary resistance by bacteria to phages. The next oldest using this phrasing, for example, is that of Erskine [46] from 1969, which refers to phage tolerance as a phage rather than bacterial property. Equivalently, in 2022 Marquioni et al. [47] describe a “phage tolerance to NaCl” and “UV light” (p. 5), and Shahin et al. [48] who a “bacteriophage tolerance to different temperatures” (p. 2), etc. See too, for example, [49,50], also from 2022. This meaning is indicated at the bottom in Figure 2.

Mathews [51] in 1970, by contrast, used “Phage tolerance” synonymously with phage genetic resistance but as a means of describing bacterial mutants that are still adsorbed by phages but which nonetheless block phage replication following that adsorption (the upper-right meaning in Figure 2), and indeed these bacteria survive the phage infection (thereby representing a form of “phage negation”, as considered in Section 2.2). Radke and Siegel [52], in their 1971 publication, similarly seem to distinguish between mutational resistance that blocks phage adsorption from a phage tolerance which involves mutational changes to bacterial functions that act after phage adsorption (p. 434): “All five [phage-resistant bacterial mutants] did not adsorb the phage, indicating that a change in the phage receptor rather than *phage tolerance* had occurred”. The latter appears to be consistent with the usage by Ito [53], in 1973, p. 7: “These mutants are able to adsorb phages but are not killed by them”. Of the four publications which Ito cites as observing bacterial mutants with similar properties, however, only that of Mathews [51] also refers to this as being an example of phage tolerance or otherwise contains the terms “tolerance” or “tolerant”. Ironically, given the usage of Mathews [51], the phage tolerance system described by Tzipilevich et al. [40], as noted above, is characterized by a lack of phage adsorption rather than a post-adsorption inhibition of phages.

From this admittedly less than comprehensive search of the literature, I conclude that a current meaning of “Phage tolerance” is to describe mechanisms of inhibition of phage action that are only temporary in their display (upper-left, Figure 2). Furthermore, many such mechanisms appear to be associated with the bacterial formation of biofilms or equivalent structures, i.e., “phage tolerance” as the term is being used here. Alternatively, the term has been used to describe bacterial mutations that result in a genetic resistance to phages that interferes explicitly with phage functioning after the virion-adsorption step, as well as being used simply to describe properties of phages. Though potentially ambiguous with its at least three general meanings, plus its occasional use as synonymous to genetic resistance, whether full or “Partial” (for example, see [54,55]), it nonetheless appears to be justifiable to employ “Phage tolerance” to describe a variety of only temporary bacterial properties that can allow them to better endure exposure either to phages, i.e., as equivalent to use of the term “tolerance”, to describe temporary mechanisms that allow bacteria to endure exposure to antibiotic (main text). Indeed, Koonjan et al. [56] explicitly make the same conceptual argument that “Perhaps the same tolerance phenomenon is exhibited by bacteria exposed to phages…” (p. 11), as so too do Ciofu et al. [31].

## 2. Phage Tolerance of Bacterial Biofilms

**Figure 3:**
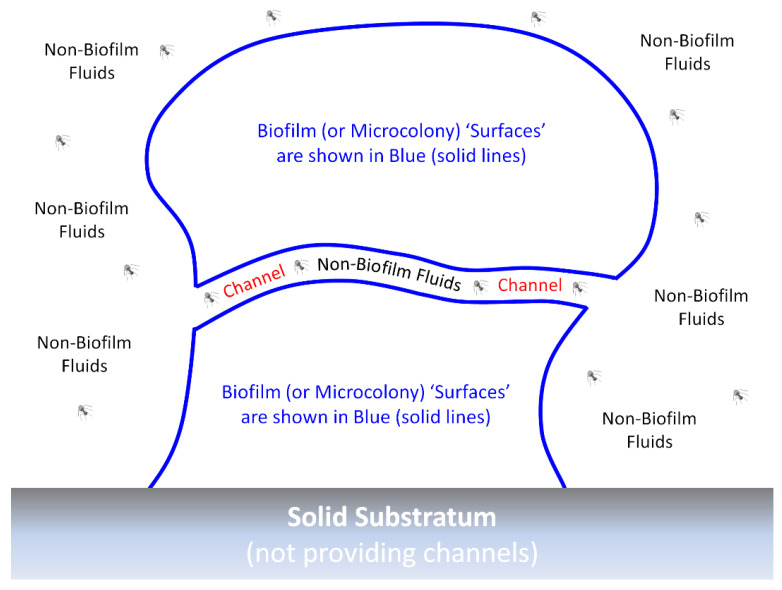
An idealized biofilm or biofilm microcolony. The “surfaces” of biofilms or microcolonies are sufficiently in contact with non-biofilm fluids that phage encounter may occur without virions penetrating much beyond a single “surface” layer of bacteria. These surfaces can include the walls of any channels that may be found within the biofilm, which would be relevant here particularly if those channels are wide enough to allow relatively easy virion passage. By analogy are various epithelial surfaces that are in contact with materials that are not body tissues, e.g., air or the contents of the gastrointestinal tract. Moreover, by analogy, such body surfaces are the primary locations of first encounter with potential pathogens, even though additional surfaces for such encounter can be created, as well, due to wounding. Actual channels—whether through biofilms, microcolonies, or bodies—would, of course, have three dimensions rather than the two dimensions depicted in the figure. Note also that biofilms can consist of individual or instead multiple microorganism species and that details of biofilm structure, such as of individual bacteria, as well as biofilm matrix filling the voids found between the blue lines, are not provided in the figure.

Bacterial tolerance of antibiotics is both typically and traditionally linked to bacteria existence within biofilms [4,8,38,57]. This tolerance can be due to poor penetration of antibiotics into biofilms as well as due to poor bacterial susceptibility to antibiotics even given sufficient antibiotic biofilm penetration. The latter phenotypic changes are associated particularly with what are known as persister variants [4,58,59], i.e., individual, otherwise antibiotic-sensitive bacteria that remain viable despite their exposure to what would be inhibiting antibiotic concentrations during MIC determinations. Here I consider instead how bacterial tolerance of phages can be manifest, particularly in association with bacterial biofilms (Figure 3).

### 2.1. Multiple Perspectives on Phage Tolerance

With phages and biofilms, we can consider similar phenomena to those limitations on antibiotic action against biofilms, as have been summarized and listed by Bull et al. [60], i.e., phage-tolerance sensu upper-left, Figure 2. These include a poor potential for phages to penetrate deeply into otherwise intact biofilms. Such a reduced virion-penetration ability may occur due to a combination of (i) potentially poor phage virion diffusion into and through biofilm matrix (though which may not be absolutely blocked even by intact matrix) and (ii) virion adsorption to bacteria found perhaps especially on the surfaces of biofilms rather than those phages first adsorbing bacteria found beneath those surfaces [39,44,61,62,63,64,65,66,67,68]. By biofilm “surface”, I mean in contact with non-biofilm fluids rather than necessarily observable visually only on the outside of biofilms, e.g., so as to include as well the linings of biofilm-associated channels [69] (Figure 3). 

Even if adsorption is not occurring to a bacterium [70,71], simply that bacterium’s bulk (or associated biofilm matrix [72,73]) may still interfere with the velocity of virion movement [74], such as the speed of a virion’s movement into the interior of a biofilm, or indeed virion access to that interior at all [72,73]. In addition, (iii) is a reduced phage infection potential of especially biofilm-buried (not-surface) bacteria once those bacteria have been reached, or perhaps more generally a lack of phage preference for more mature aspects of biofilm targets [63,75]. See equivalently, for the latter, the concept of self-organized bacterial refuges from more efficient phage predation [76]; p. 12830 of that publication states, “What is required for long-lived coexistence on the edge of bacterial refuges is merely that the bacteria in the center of the colony are so resilient that phage cannot sustain themselves in there, whereas recently divided bacteria on the edge of the colonies are (possibly very) susceptible to phage infection”.

These various mechanisms alternatively can be described ((i) and (ii)) as forms of “hiding”, “shading”, or “masking” of inner (not-surface) bacteria, i.e., as mediated by biofilm matrix or instead by outer layers of biofilm bacteria [25,63,77]. Shading mediated by biofilm bacteria (ii) also may be described as sorptive scavenging, including (1) to already lytically phage-infected bacteria [63,78]; (2) to dead but still fully intact bacteria [79] (which classically are what have been used for in vitro phage adsorption assays [26]); (3) to adsorbable but otherwise phage-resistant bacteria [72] (resulting depending on the system considered in either phage negation or bacterial self-sacrifice, both as considered in Section 2.2, though in [72] neither are presented as mechanisms of phage tolerance); (4) to metabolically quiescent bacteria [72] (more on these immediately below, as well as in Section 2.2); and (5) perhaps also phage adsorption of already-lysed bacteria [60,80]. The latter idea is a variation on the assertions of Rabinovitch et al. [81]: “Bacterial debris—an ecological mechanism for coexistence of bacteria and their viruses”, and see also [82]. Bacterial outer membrane vesicles (6) are also known to be able to adsorb phages [83,84,85] and thereby could be involved in sorptive scavenging of phage virions, as well. Given the localized higher concentration and somewhat spatially fixed nature of bacteria associated with biofilms, these outer membrane vesicles [86,87], as well as already phage-infected bacteria, dead or metabolically quiescent bacterial cells, and bacterial lytic debris, all might readily serve as anti-virion sorptive scavengers especially within biofilms.

In addition, (iii) is the noted physiological protection [25], including as potentially due to bacterial entrance into decreased growth rate, starvation, or stationary phase-like states, which seem to be able to reduce the speed of phage adsorptions, diminish phage infection activities, and/or delay phage-induced bacterial lysis [41,63,68,74,88,89,90,91,92,93,94,95,96,97,98,99,100,101,102,103,104,105,106,107,108,109,110,111]. The impact instead of reduced oxygen levels for phages infecting facultatively anaerobic bacteria, where reduced oxygen densities are often seen in the interior of biofilms [112], appears to be dependent on the phage-host system investigated, with only some displaying reduced burst sizes and one an extended latent period along with reduced adsorption rate [113]. Various mechanism that can provide protection from phages also can be displayed by bacteria in response to extracellular signaling [40,71,114,115,116,117] (see also [118], but then see [119,120], too). Different categories by which bacteria can interfere especially with phage lytic cycles are discussed in Section 2.2.

Toward antibiotics, all three mechanisms—the equivalents of shading, sorptive scavenging, and phenotypic resistance—can contribute to bacterial tolerance. This also can stem from biofilm bacteria entrance into stationary-phase-like states [57], as well as in association with quorum sensing [121]. Phage adsorption to surface bacteria, however, might be analogously replaced for certain antibiotics by antibiotic binding to the biofilm matrix. In addition, though not necessarily solely biofilm related, bacteria under osmoprotective conditions can achieve a tolerance both to certain antibiotics and to phages through a reversible loss of their cell walls [122,123].

Bacterial tolerance to phages may also be differentiated into non-mutational mechanisms that interfere with phage adsorption vs. non-mutational mechanisms that allow phage adsorption but interfere with the phage killing of bacteria. This distinction could be relevant during phage therapies, as well as ecologically, since phage virions are affected substantially differently by these different forms of tolerance. That is, poor virion diffusion into the biofilm matrix (as a form of shading), as well as poor phage adsorption to what otherwise would be phage-susceptible bacteria—both aspects of what I dub as phage avoidance in Section 2.2—can reduce the potential for virions to be inactivated relative to post-virion-adsorption blocks on the success of phage infections. See Box 2 for yet additional perspectives on biofilm tolerance of phages that would be due to shading.

Box 2 **Bacteria and obligately lytic phages replicating “happily” together?**

**Figure 4:**
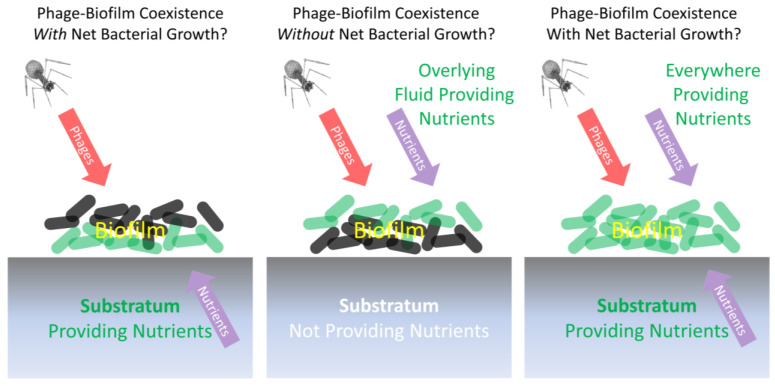
Different scenarios for biofilms limiting (left and right) or not limiting (middle) net phage exploitation despite substantial ongoing bacterial replication. Those bacteria that are more metabolically active are shown as green (bottom layer on the left, top layer in the middle, and all layers on the right), while less metabolically active bacteria are illustrated as a dark gray. If nutrients and phages come from different spatial directions (left; the Eriksen et al. [80] experimental model), then ongoing bacterial growth may be possible despite biofilm exposure to phages. If phages and nutrients come from the same direction (middle; inert substratum model), then ongoing bacterial growth may be more difficult to sustain given this phage exposure. This middle example, however, is not from Eriksen et al. [80] but instead shares similarities with the experimental model of Darch et al. [44]. We can speculate that any survival of genetically phage-sensitive bacteria in this middle example might be associated with less metabolically active stationary-phase-like bacterial physiologies (Section 2.2), thereby resulting in a lack of net bacterial growth especially as phages eliminate surface bacteria that had possessed greater access to nutrients (as considered in Box 2). If nutrients are available everywhere, all of the time (right; the Eriksen et al. [80] theoretical model), then for a microcolony to survive, it must be large enough that bacteria can replicate exponentially faster than phages can reach and infect them. Assumed in all cases is that the biofilms in question do not possess additional mechanisms of inhibition of phages that would allow net bacterial replication despite phages and nutrients reaching those bacteria from the same direction.

Fisher et al. [58] employ the term “phenotypic resistance” to describe a subset of antibiotic-tolerant bacteria that, in contrast to persister cells, are able to grow in the presence of a given antibiotic, despite those bacteria otherwise being genotypically antibiotic susceptible. This perspective is slightly reminiscent of the suggestion by Eriksen et al. [80] that (p. 337) “Continued growth from the inside of a sufficiently large colony could overwhelm [phage-mediated] killing on the colony surface”. The Eriksen et al. scenario would imply a form of shading of underlying nutrient-supplied bacteria from phages (“inside of a sufficiently large colony”) by overlying (“colony surface”) bacteria. The circumstances required for this scenario to hold may be unlikely, however, unless nutrients available to those “inside” cells (Figure 4, below, left and right examples) are supplied from a spatial direction that is different from the direction of attack by phages, as I elaborate upon in this box.

Eriksen et al.’s type of shading may be approximated if colonies, growing at or near an agar surface, were exposed to phages that were applied from above, which basically is a description of the Eriksen et al. experimental model (Figure 4, left). Alternatively, a key assumption of the Eriksen et al.’s mathematical simulation is one of a “constant nutrient level across the microcolony” (p. 338), with phages applied only to the microcolony surface (Figure 4, right). Both perspectives basically would turn a microcolony’s or biofilm’s susceptibility to phages into surface-to-volume ratio problems, with the smaller ratios of larger microcolonies favoring a failure of phages to reach all still-replicating interior bacteria, while the larger ratios of smaller microcolonies would favor that phage penetration to those still-replicating not-surface bacteria (keeping in mind that this model could be geometrically complicated by the existence of interior channels within biofilms as depicted in Figure 3).

Alternatively, to the extent that both phages and nutrients are sourced from the same spatial direction (Figure 4, middle) [44], it seems unlikely that the net growth of otherwise phenotypically phage-sensitive bacteria would be able to continue other than temporarily, i.e., since the same nutrient-replete bacteria should also be readily reached by phage virions that are diffusing along roughly the same route as nutrients. There could also be survival only of non-growing, particularly not-surface, bacteria, despite the presence of nearby phage infections (not explicitly shown in Figure 4, though discussed in its legend). The latter tolerance mechanism might provide only relatively brief protection, however, as would particularly be the case to the extent that the phage-mediated removal of overlying bacteria results in greater access by once-buried bacteria to the nutrients found in non-biofilm fluids (thereby reversing any stationary phase-like physiologies) and phages otherwise are able persist at relatively high titers in the vicinity of these now-exposed bacteria (thereby allowing phage infection of these no longer stationary phase bacteria) [42,64]. The loss of the overlying biofilm matrix also could be important toward reducing phage tolerance displayed by previously not-surface bacteria.

Note that bacterial biofilms replicating upon living or decaying tissues could very well be deriving their nutrients from “below” (left, Figure 4). In this case, especially topical phage therapies could be blunted in their impact, at least temporarily, due to a continuation of replication of phage-shaded but still nutrient well-exposed bacteria that are found adjacent to such nutrient replete substrata. This slowing of the phage impact could be even more pronounced to the extent that more mature aspects of biofilms—as potentially less easily exploited by phages [63,75]—are those both furthest from this substratum and furthest from associated nutrients, i.e., with those more mature aspects representing the biofilm surfaces that phages can most readily encounter. An example might be topical phage treatment of wound-infecting biofilms [124,125,126,127], with the phage-exposed surfaces of these biofilms in contact with air prior to phage application.

### 2.2. Summary of Bacterial Strategies of Phage Inhibition

**Figure 5:**
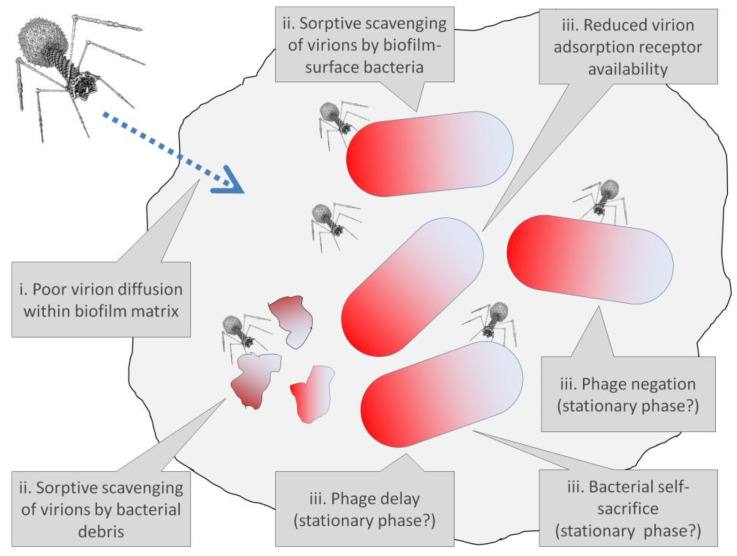
Various potential mechanisms within bacterial biofilms of tolerance of phages. Biofilm matrix is represented as a gray blob encompassing a majority of the figure, while bacteria, not drawn to scale, are represented by shaded bacilli shapes. Note that not all tolerance mechanisms necessarily involve changes in bacterial phenotypes or are associated solely with biofilm existence. For instance, sorptive scavenging, as a tolerance mechanism, is a function at least in part of bacterial spatial arrangements within biofilms rather than strictly new bacterial phenotypes, while stationary-phase-like physiologies, representing changes in bacterial phenotype, are potentially seen with planktonic bacteria, as well as by not-surface bacteria found within bacterial biofilms. Reduced receptor availability for virion adsorption similarly need not be solely associated with bacterial biofilm lifestyles. Phage avoidance, negation, phage delay, and bacterial self-sacrifice are discussed more fully in Box 3, with phage avoidance associated in the figure with either poor virion diffusion or reduced adsorption-receptor availability upon bacteria.

Overall, the bacteria-mediated inhibition of phage actions, whether associated with phage tolerance or instead with new resistance mutations, can include a failure of phages to simply reach bacteria ((i) from the previous section, and see Figure 5) or instead a failure of phages to reach more-buried (not-surface) bacteria. The latter can be a result of adsorbing either overlying bacteria (living, already phage infected, starved, or dead) or instead adsorbing bacterial lytic products ((ii) from the previous section, and again see Figure 5). Another explanation is the failure of phages to adsorb bacteria despite successfully reaching or nearly reaching those bacteria; this could be a consequence of physiological changes in bacterial phenotypes as would be included under (iii), could be due to thick capsules surrounding those bacteria [128], or could be because of bacterial production of outer membrane vesicles that can serve as adsorption decoys, i.e., as resulting in sorptive scavenging.

Bacteria under certain circumstances or at certain times also might avoid being killed, such as by otherwise obligately lytic phages, despite being both genetically sensitive to and adsorbed by those phages. This I call “negation” (Box 3), which also may be included under (iii) from the previous section (see also Figure 5) and which we can speculate may be associated in terms of phage tolerance with bacterial starvation, stationary-phase-like bacterial physiological states, or bacterial extracellular signaling. In Box 3, also discussed are what I describe as “bacterial self-sacrifice” and “phage delay” (see Figure 5 as well), both of which similarly may be manifestations of the above item (iii) to the extent that they too are associated with a tolerance of phages resulting from physiological changes to bacteria.

Box 3 **Phage avoidance, phage negation, bacterial self-sacrifice, and phage delay.**

**Figure 6:**
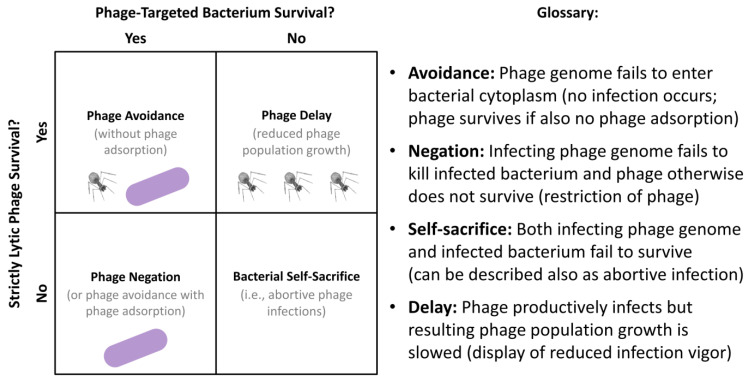
Distinguishing mechanisms of bacterial inhibition of phage activity in terms of phage vs. bacterial survival. Illustrations refer to either bacterial survival (purple bacilli as found to the far left), would-be infecting phage survival (upper-left of the 2 × 2 matrix), or some degree of successful phage amplification in numbers (upper-right also of the 2 × 2 matrix). Bacterial self-sacrifice, as defined here (lower-right, 2 × 2 matrix), results in survival of neither an infecting phage nor the phage-infected bacterium. Not shown is a productive lytic phage infection of a fully phage-susceptible bacterium, which would result in a combination of greater phage production than that seen with phage delay, for example (upper-right, 2 × 2 matrix), along with a loss of viability of the phage-infected bacterium. Categories of bacterial interference with phage infection activities and antibacterial action are defined to the right. Discussion of these mechanisms of bacterial resistance can be found in Box 3.

Mechanisms by which bacteria can interfere with phage action we can differentiate in various ways. In this box, I distinguish among them in terms of a combination of their impact on the phage-encountered bacterium and on subsequent phage-infection productivity. The lowest bacterial impact I have termed “phage avoidance”. A greater but still not very detrimental impact on bacteria I describe as “phage negation”. A substantial detrimental impact on phage-infected bacteria I describe as “bacterial self-sacrifice”. Also involving substantial detrimental impacts on individual phage-infected bacteria, particularly given lytic phage infections, but with a less than absolute impact on phage productivity is “phage delay”. I elaborate on these concepts as follow; see Figure 6 (below) for summary as well as definitions. It is important further to recognize that all four phenomena could be going on within a single phage or bacterial population given phenotypic and/or genotypic heterogeneity, e.g., with some phages failing to adsorb upon bacterial encounter (phage avoidance), others failing to kill the bacteria they are infecting (phage negation), still others killing the adsorbed bacterium but not producing phage progeny (bacterial self-sacrifice), and yet others killing the adsorbed bacterium while producing new virions, albeit potentially producing those virions at a reduced rate relative to as observed under optimal conditions (phage delay). Though these various mechanisms can be associated with bacterial resistance to phages, in this box I especially emphasize the extent to which they may be associated instead with phage tolerance. For further discussion, particularly in terms of how these various mechanisms, especially as bacterial resistance to phages, can drive bacterial evolution, see the eBook Chapters 18 [129], 19 [130], and 20 [131] of [132].

Phage *avoidance* refers to mechanisms that interfere with phage genome entrance into bacterial cytoplasms. In terms of resistance, this would be alterations of bacterial surface molecules or instead bacteria display of superinfection exclusion, with the latter being a blocking of phage genome uptake following irreversible virion attachment to a bacterial cell. In terms of tolerance, non-superinfection exclusion-type avoidance (phage avoidance without phage adsorption; upper-left of the 2 × 2 matrix, Figure 6) may be due to temporary reductions in display by bacteria of phage receptor molecules (see, for example, [133]). Alternatively, this may be due to shading of underlying bacteria by the biofilm matrix or, instead, interference with phage diffusion by bacterial glycocalyx more generally. At least a partial avoidance in terms of slower rates of phage adsorption may be seen with stationary phase bacteria or simply bacteria that are more slowly replicating, which at least in part is likely due to these bacteria being smaller in size than bacteria that are exponentially replicating [89,102,107,134].

Phage *negation* refers to mechanisms of phage inactivation that act after phage genome entrance into bacterial cytoplasms and which are associated with the survival of the phage-infected bacterium but not of the infecting phage. In terms of resistance, this traditionally has included the actions of restriction-modification systems but also can be due to superinfection immunity (see also the upper-right concept of “Phage tolerance” in Figure 2). Phage negation as phage tolerance (sensu upper-left, Figure 2) potentially might be observed given phage adsorption of bacteria possessing stationary-phase-like physiologies. At least for phage T4 of *Escherichia coli*, however, the results of Bryan et al. [111] suggest that relatively little phage negation is physiologically acquired by stationary-phase cells, though perhaps not no phage negation at all. That conclusion comes with a caveat, though, and that is that it is difficult to assess the post-adsorption genetic survival of phage-infected bacteria, as due to phage tolerance, in terms of colony-forming units without first adding nutrients to cultures, which in turn can result in a re-activation of phage infections and thus the potential loss of phage negation (i.e., resulting instead in phage-induced bacteria killing and potentially also virion production). Other studies, by contrast, have tended to look at losses of phage viability rather than declines in bacteria killing given phage adsorption of starved or stationary phase bacteria. Therefore, the extent to which bacterial starvation or entry into the stationary phase can result in significant declines in bacteria killing, given that phage adsorption still occurs, remains in my opinion underexplored.

Bacterial *self-sacrifice* refers to mechanisms that result in the killing of both the infecting phage and the infected bacterium. This likely is useful not for the individual infected bacteria that are killed but instead for the rest of clonal populations of bacteria, such as making up single-species bacterial microcolonies or biofilms, i.e., as due to phage propagation being quelled [135]. Typically these mechanisms from a resistance perspective would be viewed as consequences of the action of abortive infection systems, which as defined in a strict sense implies that a bacterium’s anti-phage activity is suicidal to the expressing cell [135]. Such suicidal behavior as tolerance might be seen given phage adsorption of bacteria displaying starvation or stationary-phase-like physiologies, i.e., should that adsorption lead to both bacterial and phage death (the latter seen as a lack of production or release of new virions).

We can at least speculate that many of the studies showing phage inactivation upon adsorption to starved or stationary-phase bacteria [91,93,94,100,101,103,111,136] are associated also with a lack of bacterial survival. Failures of phage infections of such bacteria to produce virions should be observed particularly in the absence of addition of nutrients [103], however, given the noted potential for nutrient addition to reactivate stalled phage infections [111]. Note also that the duration of infected-cell incubation prior to nutrient addition may be relevant toward distinguishing bacterial self-sacrifice (no phages produced, e.g., over shorter duration incubations during which phage infections may only partially progress toward progeny production) from phage delay (instead some phages produced perhaps over longer duration incubations). It alternatively is conceivable that an absence of added nutrients over relatively long periods might result in a transition, by at least some bacteria, from what might have been perceived as displays of self-sacrifice especially upon plating (no phages produced, and an infected bacterium does not survive) to instead phage negation (no phages produced, but an infected bacterium does survive).

Phage *delay* refers to mechanisms that slow but do not completely block phage population growth. For example, this can be associated with reductions in phage burst sizes or extensions of phage latent periods (the latter especially without corresponding increases in phage burst sizes), but also due to reduced but not eliminated rates of phage adsorption to individual bacteria [63]. This also could be dubbed as (p. 1) “Less efficient phage amplification” [30], a “Scavenger response” [111], or “Reduced infection vigor” [137] and should generally be easier to detect to the extent that new free virions are generated, e.g., during single-step growth experiments [138,139]. This comes with a caveat, however, that only a fraction of phage-infected bacteria may succeed in producing new virions during such experiments, thereby giving an impression, if not necessarily the actuality, of overall smaller burst sizes: that is, should for example most or all phage-infected bacteria produce virions upon plating (with its associated nutrient addition) but only some producing virions under the actual experimental conditions.

Phage delay in various guises seems to have been observed by multiple researchers in terms of tolerance associated with bacterial starvation, bacteria entrance into stationary-phase-like states, or as associated simply slower bacterial growth [41,88,90,95,96,97,98,99,100,102,103,105,107,108,111]. In addition, however, are declines in phage-effective burst sizes due to sorptive scavenging by already phage-infected bacteria. Such adsorption often is referred to as superinfection in the phage literature and may be more likely within the close confines of bacterial biofilms given a physical surrounding of phage-infected bacteria by other phage-infected bacteria, e.g., as Yin and McCaskill [74] allude to as possibly occurring during phage plaque growth vs. during phage exploitation of planktonic bacterial cultures. These various mechanisms of phage delay, I argue [63], could be useful to bacterial microcolonies and biofilms to the extent that bacterial dissemination from these structures might thereby be allowed to occur prior to substantial local phage propagation, i.e., as due to delays in that phage propagation.

There is also a concept described as pseudolysogeny [109,110,140,141], which explicitly is a delay in the development of phage infections of starved bacteria but as resulting, potentially, in new-phage production once nutrients are supplied. Bryan et al. [111] describe an equivalent phenomenon associated with phage infection of stationary phase bacteria as hibernation. Either differs from phage delay to the extent that phage infections are stalled, that is, not producing phage progeny until the surrounding environment has been modified, i.e., as modified especially upon supply of additional nutrients to bacteria. These phenomena also differ from phage negation in that the infecting phage continues to survive; they also differ from bacterial self-sacrifice in that so too does the infected bacterium continue to survive, in both cases as observed prior to the supplying of additional nutrients to cultures. Phage negation, bacterial self-sacrifice, and phage delay thus all should be viewed as results of phage infections that occur over the course of more or less constant physiological conditions, whereas with improvement of these conditions then pseudolysogenic or hibernating phage infections may be reactivated to produce phage progeny. The resulting phage amplification, however, will not necessarily occur with the same vigor as may be observed given phage infection of exponentially replicating bacteria.

Whether due to tolerance or resistance, phage ecological productivity is eliminated, by definition, by both phage negation and bacterial self-sacrifice (bottom row of the 2 × 2 matrix, Figure 6), but only incompletely by phage delay and potentially not reduced given a display of phage avoidance by bacteria (top-row of the 2 × 2 matrix, Figure 6). The latter would be the case if phage virion adsorption does not immediately occur but could still occur to a somewhat phage-susceptible bacterium sometime in the future, thereby resulting also in a phage delay—in that case, though, a delay in terms of rates of phages finding bacteria to infect rather than in terms of reduced infection productivity following phage adsorption. Bacterial ecological productivity, on the other hand, would be retained, again by definition, given either phage avoidance or phage negation (left column of the 2 × 2 matrix, Figure 6). The viability of individual phage-infected bacteria, however, would be lost given either bacterial self-sacrifice or phage delay (right-column, 2 × 2 matrix, Figure 6).

### 2.3. Phage Tolerance as Ecological Phenomena

All mechanisms of tolerance, whether countering antibiotics or instead countering phage actions, represent *ecological* phenomena. That is, they are mechanisms that (1) represent changes in organism phenotype as a function of environmental conditions, such as existing within a bacterial biofilm rather than experiencing planktonic growth; (2) involve an interaction of an organism with its environment (bacteria interacting either with phages or with antibiotics); and, as considered here, (3) are not dependent in their acquisition, at least in the near term, on changes to organism (bacterial) genotype.

The latter point means, in particular, that the tolerance phenotypes under discussion are not displayed in association, also in the near term, with substantial changes in allele frequencies within displaying bacterial populations, and nor is this tolerance associated with a genetically distinct subset of a bacterial population that is being exposed to either phages or antibiotics. Tolerance, from this perspective, means instead that a genetically homogeneous bacterial population can be both sensitive and not sensitive, or least less sensitive, to a phage population or to an antibiotic treatment. That sensitivity vs. reduced sensitivity, however, is observed under what are potentially different ecological circumstances, such as planktonic vs. biofilm-associated, respectively. 

Otherwise, but not addressed here, is the extent to which persister cells displaying antibiotic tolerance also may tend to be phage tolerant or whether phages can be used to target true persister cells during biofilm treatments [142,143,144], i.e., genetically antibiotic-sensitive bacteria that antibiotics are not able to eliminate but phages might be able to kill. Rather, the prevailing suggestion is that certain aspects of especially bacterial biofilms can result in some degree of bacterial tolerance of phage attack and, furthermore, whether to phages or instead to antibiotics, tolerance is an ecological rather than necessarily also a near-term evolutionary phenomenon. Contrast instead the acquisition of resistance to phages by previously genetically phage-sensitive bacteria, which explicitly *is* an evolutionary phenomenon [145], as is addressed in the following section especially from a perspective of phage interactions with bacterial biofilms.

## 3. Phage Resistance and Bacterial Biofilms

Evolution can be defined as a change in the frequency of one or more alleles over time as this occurs within a single organism population. Prominent evolutionary mechanisms—such as those giving rise to changes in the frequency of alleles that bestow resistance to phages or antibiotics—are mutation and natural selection. Resistance systems also may enter bacterial populations from different bacterial populations in the course of genetic migration/horizontal gene transfer, such as via acquisition of mobile genetic elements [146,147]. The latter, though, are not an emphasis here. Moreover, phage mutation to overcome bacterial resistance to phages is also not addressed [148,149,150,151], i.e., as can result, in association with bacterial resistance evolution, in what is known as antagonistic coevolution [132,150]. Phage treatment, in combination with antibiotics, should in many cases result in reduced resistance evolution relative to either agent acting alone [152,153,154,155,156,157,158,159] at least absent cross resistance [160,161,162]; however, that phenomenon, too, is not emphasized here. Instead, in this section we consider bacterial mutation simply to phage resistance and especially how the frequency of resulting phage-resistant bacterial mutants can be impacted by bacterial existence within biofilms, though with Section 3.1.1 and Section 3.3 both addressing more general issues of bacterial mutation to phage resistance. Molecular mechanisms of bacterial resistance have been explored and summarized in numerous articles, e.g., [135,149,163,164,165,166].

### 3.1. Mutation, Selection, and Antagonistic Pleiotropies

#### 3.1.1. General Considerations

Resistance mutations [167], as well as the acquisition of resistance systems by horizontal gene transfer, generally are considered to arise independently of exposure to whatever it is that is being resisted. Particularly in terms of resistance mutations, this means that the initial degree of presence of those mutations within a naïve population will, in absolute terms, be a function of a combination of rates of mutation to resistance and the size of the population in which the mutations are arising (and also, for smaller populations and lower mutation rates, simply chance [167]). Thus, small populations—especially ones with low mutation rates to resistance—will, relative to larger populations, both carry fewer mutation types and have a lower potential to carry any resistance mutations at all. Mutations to resistance therefore will tend to always be present within sufficiently large naturally occurring bacterial populations, whereas the potential for those mutations to be present, e.g., such as in the individual wells of 96-well microtiter plates, can be less likely (see Section 21.3 of Chapter 21 [168] of [132] for a brief essay discussing the latter, as well as [30,68]). As a consequence it can be crucial, in studies exploring the occurrence of phage resistance in the presence of phages, for the total numbers of treated bacteria and/or for bacterial concentrations, along with experimental volumes, to be reported. Whether or not bacterial mutations to phage resistance are easily detected, however, will depend on the second prominent evolutionary mechanism controlling the frequency of alleles, as mentioned above, i.e., natural selection. 

**Figure 7:**
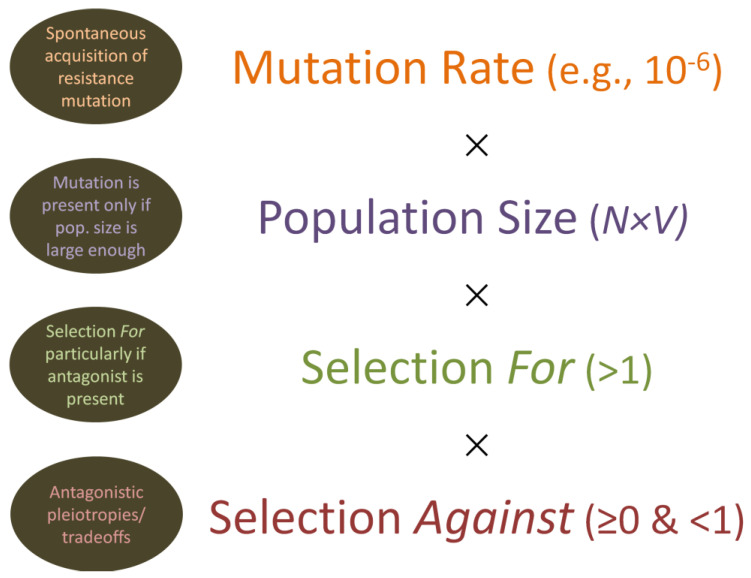
Mutation to resistance, along with selection for and against those mutations. Mutations are assumed to occur spontaneously at some rate per bacterial generation, can be somewhat lower or higher in frequency than the indicated 10^−6^ (e.g., 10^−7^, 10^−8^, or 10^−5^), and will tend to occur independently of any selecting force. For “Population size”, *N × V* refers to bacterial concentration (*N*) multiplied by the volume (*V*) of the occupied environment. Resistance mutations, by definition, are selected *for* when the selective agent is present. These same mutations, however, can also be detrimental to the functioning of the otherwise resisting organism, resulting also in some degree of selection *against* (i.e., as due to antagonistic pleiotropic effects, also known as tradeoffs). If selection *for* is stronger than selection *against*, then the resistance allele should increase in frequency within a population as long as the selecting agent is present. Otherwise, the resistance allele typically, though not always, should decrease in frequency as a consequence of selection against. Note that selection against, in Darwinian fitness terms, is equal to zero if the resistance allele is lethal to carrying individuals. Note otherwise that the timeline of both mutation and selection is not explicitly indicated in the illustration.

There are two key mechanisms by which natural selection will tend to impact the frequency of resistance alleles. These can be referred to simply as selection *for* vs. selection *against* these alleles (Figure 7). Selection for is seen predominantly when the agent being resisted is present in the environment, and particularly when that agent is present at sufficiently high concentrations, whereas selection against has to do with what are known as antagonistic pleiotropies, which also are known as tradeoffs. That is, a mutation to resistance may be harmful to other important aspects of organism functioning, such as in terms of the virulence of bacterial pathogens [169,170,171]. The result can be various degrees of selection against resistance mutations, with this selection against typically most noticeable particularly when the selecting agent, such as phages or antibiotics, is not highly prevalent in a bacterial population’s environment. 

Given the above considerations, we can describe a mutation-selection balance, with mutations inputting resistance alleles into a bacterial population at some rate and, especially given antagonistic pleiotropic effects, selection reducing the frequency of those alleles also at some rate, with the latter being most evident when the agent being resisted is absent. At an extreme in terms of tradeoffs, resistance mutations may be lethal to the harboring organism, resulting in near instantaneous removal of those alleles from populations and thereby a perceived lack of potential for a given bacterial strain to mutate to resistance to a particular phage. See Section 25.5.3 of Chapter 25 [151] of [132] for further discussion of the latter point, as well as Chapter 22 [172] also of [132].

#### 3.1.2. Taking Biofilms into Account

What this has to do with phages and biofilms is two-fold. First is that, just as with planktonic populations of bacteria, whether bacterial mutations to phage resistance are present—particularly in the absence of selecting phages—or to what degree they are present in terms of frequency will be a function of a combination of bacterial mutation rates to phage resistance, the size of a bacterial population, and the impact of natural selection regarding pleiotropic antagonistic effects. It is possible, though, that bacterial mutation rates will be higher within biofilms than within planktonic populations [38,57,173], including due to oxidative stress [174,175,176] but also potentially as associated with low oxygen conditions [177]. Mutations may also accumulate to higher levels in biofilms vs. planktonic cultures even without mutation rates being higher and in spite the existence of antagonistic pleiotropies; this is due to lower strengths of natural selection, particularly selection against acting within spatially structure environments [178]. Those various mechanisms should serve to counter an argument that bacterial mutations to phage resistance should accumulate especially among those biofilm bacteria that are most rapidly replicating [76]; however, mutation to resistance among those bacteria could very well be common as well. 

Selection for phage resistance could be strongest among the same rapidly replicating bacteria if those bacteria are also most easily reached by phages [80]. This greater phage access, particularly to more rapidly replicating surface bacteria, appears to have been demonstrated by Testa et al. [68] in terms of colonies growing upon agar surfaces. Bacterial population sizes also might be larger, or instead smaller, within biofilms vs. planktonic cultures, and particularly smaller within mixed-species or mixed-strain biofilms than in mono-species biofilms assuming overall numbers of cells remain constant [68]. Lastly, it is possible that there will be differences in terms of the strength of selection against resistance alleles, again as antagonistic pleiotropies, as bacteria are found within biofilms vs. as planktonic cultures. For example, a mutation might be more detrimental (stronger selection) during active biofilm formation, and this could be so even if the strength of natural selection acting against that mutation within an already established biofilm might be lower [178]. Particularly, the strength of natural selection against phage resistance could be weaker especially in association with well-established not-surface bacteria due to reduced metabolic demands being placed on those bacteria vs. biofilm-surface or planktonic bacteria. In short, the dynamics of bacterial resistance mutation accumulation could very well be different in biofilms relative to within well-mixed planktonic cultures, but there likely exist multiple mechanisms giving rise to those differences, including in terms of the strength as well as efficiency of selection for and also against that resistance (Table 1).

### 3.2. Reduced Selection Efficiency for Resistance within Biofilms

As hinted at in the previous paragraph, as well as in Table 1, a key distinction between planktonic and biofilm-associated populations of bacteria can be the degree of heterogeneity of their exposure to selecting phages [36,80]. That is, in planktonic cultures, particularly ones which are well mixed, phages should be able to reach all of the bacteria present with equivalent likelihood. This, for a given phage virion concentration, should result in an extreme of efficiency of selection for resistance alleles, assuming that those alleles are both present and not pleiotropically lethal to the carrying bacteria. In other words, given sufficient, i.e., “Inundative” [37] phage concentrations, there is a potential for bacterial mutations to phage resistance to rise to fixation relatively rapidly within especially well-mixed planktonic cultures, where “fixation” is defined as when the frequency of an allele within a population essentially is equal to 1.0, with thereby few or no phage-sensitive bacteria remaining. Such a high efficiency of selection assumes, of course, that the bacterial population is also homogeneous phenotypically rather than some but not all bacteria displaying a physiological tolerance of phages; the latter would have the effect of reducing the strength of natural selection for resistance acting on those phage-tolerant bacteria.

Thus, within biofilms the ability of phages to reach specific bacteria should vary depending on the micro-location of the bacteria. This especially may be the degree to which bacteria are directly in contact with non-biofilm fluids (surface bacteria; Figure 3) vs. found sufficiently buried within biofilms (not-surface bacteria) that they are far from those fluids and thereby shaded by biofilm material; bacteria existing within “ephemeral spatial refuges” are equivalent (p. 7) [30]. This means, as noted, that selection for resistance alleles in a biofilm context should be less efficient than selection for equivalent alleles within planktonic bacterial populations, i.e., since not all of the biofilm bacteria present will be exposed to the same extent to selecting phages. Phage tolerance associated with only a fraction of individual bacteria—particularly physiologically, resulting in either phage avoidance or phage negation (Figure 6)—similarly should reduce the efficiency of selection for resistance alleles across a bacterial population residing within biofilms relative to planktonic cultures, as may have been the case, for example, given *Vibrio anguillarum* exposure to phage KVP40 [62]. That is, inhomogeneities in the strength of selection for resistance, particularly reduced strength of selection in some bacteria, e.g., due to tolerance or equivalently because phages have difficulty reaching some but not other bacteria, should reduce the efficiency of selection for resistance.

Overall, then, reduced efficiency of selection for resistance is expected the fewer bacteria within a population that phages are able to negatively impact [179]. Furthermore, given the various mechanisms discussed, there should exist a potential for resistance alleles to be net selected *for* in one portion of a single biofilm (that portion most exposed to phages and/or displaying less tolerance), while, at the same time, the same alleles may be net selected *against* in other portions of the same biofilm, with the latter again being due to antagonistic pleiotropies.

### 3.3. Community Resistance vs. Treatment Resistance

An additional consideration regarding bacterial resistance to phages is exactly when—over both shorter and longer time spans—resistance has risen within a bacterial population to some approximation of fixation. In particular, is this prior to exposure of a bacterial population to a given phage type or instead following that exposure? Above we have considered only the latter scenario, i.e., where an otherwise naïve bacterial population is exposed to a new phage type and this results in selection for initially rare phage-resistance alleles in that population, assuming that the costs of carrying those alleles (as due to antagonistic pleiotropies) are not so substantial. This scenario, within a phage therapy context, can be dubbed as the development of a *treatment* resistance. As described in Dąbrowska and Abedon [180], this explicitly is resistance that rises to or toward fixation during phage treatments rather than a resistance that had risen to fixation prior to phage treatment. 

In contrast to treatment resistance is what we dubbed *community* resistance [180]. “Community” here refers to geographically localized groups of humans. Somewhat synonymous with this concept of “community resistance”, Amábile-Cuevas [181] uses the phrasing “Ancient resistance”. In either case, this is resistance that has risen to fixation within a bacterial population independent of subsequent phage treatments. This could be a consequence of past environmental exposure of bacteria to phages [182] or instead could be a consequence simply of increases in the frequency of resistance alleles within a bacterial population for reasons that are independent of phage exposure. The latter, by the way, could also be dubbed as a pleiotropy, though not an antagonistic pleiotropy, should some new allele be beneficial to the carrying bacterium in the absence of selecting phages and also happen to provide some phage resistance.

The relatively narrow host ranges of many phages [137,183] means that treatment resistance to phages, contrasting with treatment resistance to more broadly acting antibiotics, should tend to be selected for within fewer bacterial populations over the course of phage therapies. Ideally, in fact, directly phage-affected populations will be limited to just the targeted bacterial species. This suggests, if nothing else, that the use of phage therapies should not lead to the widespread evolution of phage resistance across bacterial communities, with “community” in this latter case being an ecological term referring to multiple species occupying a single locality. Consequently, phage therapies in and of themselves should not substantially increase the potential for acquisition of phage-resistance alleles via horizontal gene transfer by phage-targeted bacterial pathogens, contrasting antibiotics for which the dynamics of resistance evolution and acquisition can involve much more than just individual bacterial species [181,184,185]. On the other hand, phage-induced bacterial lysis could promote horizontal gene transfer from phage-targeted bacteria to other, not-targeted bacterial species [186,187].

## 4. Conclusions

It seems clear that we are trending toward the use of “tolerance” to describe mechanisms of only temporarily—and not a direct consequence of recent bacterial genetic change—reduced bacterial susceptibility to phages, particularly as displayed by biofilms (Box 1). This tolerance can occur via a number of different general mechanisms (Box 2 and Box 3), again as associated especially with bacterial biofilms. Here we have explored various facets of the bacterial tolerance of phages vs. bacterial resistance to phages. It is important to keep in mind, though, that while both are ecological phenomena, as they affect phage interactions with bacteria, only phage resistance, such as may be selected for in the course of phage therapies, is an immediate consequence of bacterial evolutionary change. Thus, with tolerance, as considered here, both phage and antibiotic therapies can fail without bacterial evolution occurring, i.e., as a consequence of ecological factors alone. Treatment failures of course can occur also as a consequence of bacterial evolution of resistance, though only to the extent that negative impacts of antagonistic pleiotropies associated with new resistance phenotypes are not too extreme [188]. With tolerance mechanisms, by contrast, no such new antagonistic pleiotropies should permanently exist, as the tolerance phenotypes described should by definition be reversible.

An obvious means of addressing resistance, whether treatment resistance or community resistance, is to incorporate into therapies new phages with new host ranges [189]. It may be more involved, however, to modify treatment approaches to address the many aspects of phage tolerance [36]. Possible approaches to combating phage tolerance, though, may involve (1) improving phage penetration into the biofilm matrix, e.g., by supplying matrix-degrading enzymes [13,14]; (2) employing phages that are better equipped to kill, lyse, and propagate on stationary phase bacteria [36,103,190,191,192]; (3) using multiple dosings of phages to sustain adequate phage titers in the vicinity of targeted bacteria over time [193]; (4) perhaps also treating with phages targeting a diversity of receptor molecules should the expression of some but not all of these receptors be downregulated; and (5) treating with a diversity of phages also because some phages may, for so-far unknown reasons, be better equipped at overcoming tolerance under different circumstances than other phages. Ultimately, though, efforts toward increasing phage therapy successes [194] should necessitate distinguishing phage tolerance from phage resistance as root causes of treatment failures.

## Figures and Tables

**Table 1:** Reduced strength and efficiency of selection for and against resistance within biofilms *.

Reduced in Biofilms	Explanation for the Reduction
Efficiency of selection against resistance	Not-surface biofilm bacteria should display less growth relative to surface or planktonic bacteria, reducing overall especially reduced-growth-rate pleiotropic costs of phage resistance
Strength of selection against resistance	Negative impacts of antagonistic pleiotropies could be fewer for not-surface biofilm bacteria due, e.g., to lower metabolic demands relative to within growing planktonic cultures
Efficiency of selection for resistance	Not-surface biofilm bacteria may be less readily reached or affected by phages relative to surface or planktonic bacteria, resulting in less phage impact on bacterial population fitness
Strength of selection for resistance	To the extent that phage-sensitive bacteria are less impacted by phages that reach them, e.g., such as due to reduced receptor display, then being resistant also should be of lower benefit

* Strength of natural selection is as measured on an individual-bacterium basis—phage-resistant relative to phage-sensitive genotypes—given existence within a genetically non-homogenous bacterial population; that is, how much does being resistant impact a bacterium’s individual fitness? Efficiency of selection is measured across a genetically but not necessarily phenotypically or environmentally homogeneous bacterial subpopulation. For instance, do the fitness benefits of being resistant vary across a bacterial subpopulation, particularly with some bacteria benefiting less than others?

## Data Availability

Not applicable.
